# *Ferocactus herrerae* Fruits: Nutritional Significance, Phytochemical Profiling, and Biological Potentials

**DOI:** 10.1007/s11130-022-01007-9

**Published:** 2022-08-30

**Authors:** Passent M. Abdel-Baki, Rana M. Ibrahim, Nariman E. Mahdy

**Affiliations:** Pharmacognosy Department, Faculty of Pharmacy, Cairo University, Kasr-El-Ainy Street, Cairo, 11562 Egypt

**Keywords:** *Ferocactus herrerae*, Headspace, HPLC, Antioxidant activity, COX inhibition, Acetylcholinesterase inhibition

## Abstract

The current study reports for the first time the nutritional, fruit volatiles, phytochemical, and biological characteristics of *Ferocactus herrerae* J. G. Ortega fruits. The nutritional analysis revealed that carbohydrate (20.6%) was the most abundant nutrient followed by dietary fibers (11.8%), lipids (0.9%), and proteins (0.8%). It was rich in vitamins, minerals, essential, and non-essential amino acids. Gas chromatography-mass spectrometry (GC-MS) analysis of the headspace-extracted volatiles showed that 3-methyl octadecane (35.72 ± 2.38%) was the major constituent detected. Spectrophotometric determination of total phenolic and flavonoid contents of the fruit methanolic extract (ME) showed high total phenolic [9.17 ± 0.87 mg/g gallic acid equivalent (GAE)] and flavonoid [4.99 ± 0.23 mg/g quercetin equivalent (QE)] contents. The ME was analyzed using high-performance liquid chromatography with ultraviolet (HPLC-UV), which allowed for both qualitative and quantitative estimation of 16 phenolic compounds. Caffeic acid was the major phenolic acid identified [45.03 ± 0.45 mg/100 g dried powdered fruits (DW)] while quercitrin (52.65 ± 0.31 mg/100 g DW), was the major flavonoid detected. *In-vitro* assessment of the antioxidant capacities of the ME revealed pronounced activity using three comparative methods; 2, 2-diphenyl-1-picrylhydrazyl (DPPH) (132.06 ± 2.1 *μ*M Trolox equivalent (TE) /g), 2,2′-azino-di(3-ethylbenzthiazoline-6-sulfonic acid (ABTS), (241.1 ± 5.03 uM TE/g), and ferric reducing antioxidant power (FRAP) (258.9 ± 1.75 uM TE/g). Besides, remarkable anti-inflammatory [COX-1 (IC_50_ = 20.2 ± 1.1 *μ*g/mL) and COX-2 (IC_50_ = 9.8 ± 0.64 μg/mL)] and acetylcholinesterase inhibitory (IC_50_ = 1.01 ± 0.39 mg/mL) activities were observed. Finally, our results revealed that these fruits could be used effectively as functional foods and nutraceuticals suggesting an increase in their propagation.

**Supplementary Information:**

The online version contains supplementary material available at 10.1007/s11130-022-01007-9.

## Introduction

Fruits and vegetables are an essential source of nutrition and medicine for humans. Rapid advances in nutrition, medicine, and plant biotechnology have profoundly altered and revolutionized concepts of food, health, and agriculture. Nutraceuticals, nutritional therapy, phytonutrients, and phytotherapy are some of the new concepts that have emerged because of this development [1]. They are packed with a range of nutrients and bioactive compounds, including phytochemicals, vitamins, minerals, antioxidants, and many phytonutrients, which are essential for improving our health [2]. Therefore, their consumption has been reported to lessen the prevalence of numerous oxidative stress-related disorders such as cancer, cardiovascular disease, and chronic inflammatory diseases [3].

Cacti are a large and diverse group of succulent species that belong to the Cactaceae family [4]. They are commonly cultivated for ornamental purposes; however, many are planted as agricultural and industrial crops such as animal feed, vegetables, and fruits [5]. Cacti are widely used in food applications due to their reputed nutritional value [6] as well as the presence of natural pigments like betalains, which are used as safe food colorants [7]. In addition, they exhibit a variety of biological potentials, such as analgesic, anti-inflammatory, anticancer, antimicrobial, antioxidant, and hallucinogenic properties [8, 9]. *Ferocactus* is a small genus in the Cactaceae family. It comprises about 30 species of barrel-shaped cacti [4]. They are native to Mexico and America [10]. Some *Ferocactus* species are used to make cactus candy, while others are consumed like lemons and limes [10]. *F. herrerae* seeds are used to make tortillas. Its raw flesh is consumed owing to its benefits in reduction of high blood pressure [11]. The ethanolic extract of *F. herrerae* plant showed chemopreventive activity [12]. *F. echidne* has been used in silver nanoparticle synthesis due to its powerful reducing properties [13]. *F. histrix* is regarded as a potent antioxidant due to its high phenolic content [14]. The chloroformic extracts of *F. latispinus* and *F. histrix* fruits exhibited significant anti-hyperglycemic and anti-hyperlipidemic effects [15]. Phytochemical investigation of the stem extracts of *F. gracilis*, *F. pottsii*, *F. herrerae*, *F. horridus*, *F. glaucescens*, and *F. emoryi* reported considerable amounts of polyphenols in their extracts as well as potential anticancer, antibacterial, and antifungal activities [16]. *F. glaucescens* fruits have been demonstrated to be abundant in nutrients and antioxidants, making them highly recommended for human consumption [2]. According to a recent review conducted by Ramírez-Rodríguez et al., *Ferocactus* species are regarded as research opportunities because their nutritional and phytochemical characterization have not been fully described despite their nutritional and economic significance [4]. In addition, as far as we know, nutritional, phytochemical, and biological profiles of *F. herrerae* J. G. Ortega fruits are still uncovered. Thus, this study aimed to explore the nutritional properties, fruit volatiles, phytochemical constituents, antioxidant, anti-inflammatory, and acetylcholinesterase inhibitory activities of *F. herrerae* ripe fruits for the first time.

## Material and Methods

The material and methods section is reported as Supplementary online resource.

## Results and Discussion

### Nutritional Properties

The amount of total soluble solids (TSS) in the ripe fruits was 14.70 °Brix ±0.23, which was a little higher than that reported for *F. glaucescens* ripe fruits (13.5 °Brix) [2] and *F. histrix r*ipe and unripe fruits (5.30 ± 0.03 to 12.72 ± 3.19 °Brix) [14], indicating a higher content of sugars and other dissolved solids.

Regarding the pH (4.60 ± 0.23), the results showed that the ripe fruits are slightly acidic. These results are in agreement with previous reports for *F. glaucescens* ripe fruits (pH 4.7) [2] and *F. histrix* ripe and unripe fruits (pH 3.56 ± 0.28 and 2.46 ± 0.10, respectively) [14]. As a result, incorporating *F. herrerae* fruits into the human diet is both safe and beneficial, as pH 4.6 causes a decrease in plaque formation as well as it is low to induce enamel erosion [17].

The crude fiber content of the fruits was 11.80 ± 0.23 g/100 g of the dried powdered fruits (DW), higher than that detected in *F. glaucescens* ripe fruits (10.10 g/100 g DW) [2] and that of *F. peninsulae* plant (1.10 ± 0.3 g/100 g DW) [18]. Therefore, *F. herrerae* fruits could be regarded as a good source of dietary fibers.

The ripe fruits showed relatively high total carbohydrates representing 20.60 ± 0.55 g/100 DW. Analysis of the free sugars revealed the presence of glucose, fructose, and sucrose corresponding to 3.14 ± 0.23, 4.67 ± 0.52, and 0.65 ± 0.23 g/100 g DW, respectively. The high content of glucose and fructose indicates the presence of marked invertase activity that could have decreased its sucrose content [19]. The total carbohydrates and free sugar contents determined were higher when compared with those detected in *F. glaucescens* ripe fruits [2].

In addition, appreciable amounts of vitamin C [(712.33 ± 0.56 mg/100 g of the fresh fruits (FW)], E (3720.02 ± 0.21 IU/100 g FW), and provitamin A (2100.45 ± 0.12 IU/100 g FW) were detected in the ripe fruits. The vitamins detected were found to be higher than those reported in *F. glaucescens* ripe fruits in a study conducted by El-Hawary et al. [2]. The detected vitamins are proved to have antioxidant activity [20].

The results revealed that *F. herrerae* ripe fruits could be considered a rich source of micro- and macro-minerals. Among the macro-minerals, calcium predominated (50.29 ± 0.98 mg/100 g DW), having important metabolic functions as it is a component of bones and teeth, involved in blood coagulation and intracellular communication [21]. A considerable amount of magnesium was detected (9.73 ± 0.10 mg/100 g DW) which is important for muscle relaxation and in control of acid-base, water, and salt balance in the body. Sodium ion was present in a relatively high amount (33.64 ± 0.32 mg/100 g DW) which is responsible for maintaining muscle contractility by regulating the fluid volume outside the cells. Regarding the micro-minerals, iron was detected in the highest amount (2.705 ± 0.78 mg/100 g DW) which serves in the body as oxygen transport, electron transport, and prevents microcytic hypochromic anemia [22]. The other detected micro-minerals, zinc (0.108 ± 0.11 mg/100 g DW), copper (0.20 ± 0.09 mg/100 g DW), and manganese (0.076 ± 0.08 mg/100 g DW) have antioxidant potential as well as enhance the immune system [21]. The macro-minerals detected were found to be higher than those reported for *F. glaucescens*, except for magnesium [2]. The micro-mineral analysis differs to some extent from the analysis conducted for *F. glaucescens* previously [2].

The ripe fruits showed low lipid (0.9 ± 0.11 g/100 g DW) and protein contents (0.8 ± 0.15 g/100 g DW). The lipid and protein contents were lower than those of *F. glaucescens* ripe fruits (1.3 and 1.2 g/100 g DW, respectively) [2] and the pulps without spines of *F. peninsulae* plant (5·8 ± 0·8 and 8·2 ± 0·8 g/100 g DW, respectively) [18].

Moreover, the fruits contain a variety of essential and non-essential amino acids (Table 1). Phenylalanine was the major essential amino acid determined (0.55 ± 0.12 mg/g DW). Concerning the non-essential amino acids, arginine predominated corresponding to 0.99 ± 0.11 mg/g DW. Nearly the same amino acids were characterized in *F.* *glaucescens* ripe fruits, yet both vary in each amino acid concentration [2].

**Table 1:** Amino acid composition of *F. herrerae* J. G. Ortega ripe fruits

Essential amino acids	*Conc. (mg/g DW ± SD)	Non-essential amino acids	*Conc. (mg/g DW ± SD)
Histidine	0.06 ± 0.03	Alanine	0.18 ± 0.08
Isoleucine	0.12 ± 0.09	Arginine	0.99 ± 0.11
Leucine	0.20 ± 0.11	Glutamic acid	0.25 ± 0.18
Lysine	0.12 ± 0.09	Glycine	0.76 ± 0.13
Phenylalanine	0. 55 ± 0.12	Proline	0.002 ± 0.02
Threonine	0.38 ± 0.10	Serine	0.67 ± 0.11
Valine	0.12 ± 0.08	Tyrosine	0. 38 ± 0.12

*Average concentration of three replicates, DW: dry weight, SD: standard deviation

As we have noted, these fruits have high fiber, carbohydrate, mineral content, essential and non-essential content, and low lipid content, which makes them a valuable nutritious food alternative for various at-risk populations, regarding obesity and metabolic syndrome. It is worth noting that this is the first study regarding the nutritional significance of *F. herrerae* fruits.

### GC-MS Analysis of Headspace-Extracted Volatiles

The volatile constituents produced by the plants are regarded as important secondary metabolites and are extensively used in food industry as flavoring agents and preservatives [23]. In this respect, the volatiles were extracted from the ripe fruits by headspace (HS) technique coupled with GC-MS analysis (Fig. S1). As a result, 12 compounds were detected for the first time in the genus *Ferocactus* representing 95.04% of the total detected volatile components. The identified constituents belonged to various classes (Table 2), with five monoterpene hydrocarbons (14.29%), two oxygenated monoterpenes (0.78%), two alcohols (27.45%), one ester (0.13%), one hydrocarbon (35.72%) and one fatty acid (16.67%) were identified. The main constituents detected in the fruits were 3-methyl octadecane (35.72%), octadecanoic acid (16.67%), and 1-penten-3-ol (15.76%). The identified compounds differ from those characterized as unique constituents in *F cylindraceus* [24]. Thus, they could be considered as marker constituents for quality control purposes of *F. herrare* fruits. That said, to the best of our knowledge, the present report is the first on the volatile composition of *F. herrerae* fruits.

**Table 2:** Components identified in the headspace-extracted volatiles of *F. herrerae* ripe fruits

Serial	Identified component	M^+^	Rt (min)	RI	*Relative% ± SD	Chemical class
1	1-Penten-3-ol	86	5.12	720	15.76 ± 2.31	Alcohol
2	*β*-Pinene	136	17.85	982	1.70 ± 0.21	Monoterpene HC
3	Ethyl hexanoate	144	19.10	998	0.13 ± 0.11	Ester
4	*p*-Cymene	134	19.82	1029	4.36 ± 0.22	Monoterpene HC
5	Limonene	136	20.88	1034	1.56 ± 0.45	Monoterpene HC
6	*β*-Phellandrene	136	21.79	1035	3.62 ± 0.89	Monoterpene HC
7	Benzyl alcohol	108	23.56	1038	11.69 ± 2.10	Alcohol
8	*β*–Ocimene	136	25.12	1045	3.05 ± 1.45	Monoterpene HC
9	*β*-Linalool	154	26.60	1075	0.65 ± 0.12	Oxygenated monoterpene
10	*α*-Terpineol	154	27.11	1178	0.13 ± 0.11	Oxygenated monoterpene
11	3-Methyl octadecane	268	29.97	1846	35.72 ± 2.38	HC
12	Octadecanoic acid	284	30.16	2174	16.67 ± 0.91	Fatty acid
Total identified components (%)						95.04

*Average relative percentage of three replicates; HC: hydrocarbon; M^+^: molecular ion, R_t_: Retention time; RI: retention index (Kovat’s index); SD: standard deviation

### Spectrophotometric Determination of Phenolic Compounds

Many bioactivities have been linked to phenolic compounds such as phenolic acids and flavonoids including antioxidant, anticarcinogenic, cardioprotective, anti-inflammatory, and antimicrobial activities [16, 25]. As a result, the polyphenolic and flavonoid contents of *F. herrerae* J. G. Ortega ripe fruits were determined spectrophotometrically and by HPLC-UV analysis (Fig. S2 and S3). The TPC of the ME was estimated as 9.17 ± 0.87 mg/g gallic acid equivalent (GAE). While, TFC was calculated as 4.99 ± 0.23 mg/g quercetin equivalent (QE). The results were in agreement with those of *F. glaucescens* ripe fruits (TPC 8.39 ± 0.074 mg/g GAE and TFC 3.82 ± 0.019 mg/g QE) [2]. However, the TPC was higher than that of *F. histrix* unripe fruits (TPC 0.44 ± 0.19 mg GAE/ g) [14]. After all, polyphenols content in plant tissue is very critical. They act as antioxidants with protective effects against cancer, Alzheimer’s, and cardiovascular diseases [26].

### Quantitative Determination of Phenolic Compounds by HPLC-UV

HPLC-UV analysis revealed that the fruits were rich in phenolic compounds. Totally, 16 phenolics (8 phenolic acids and 8 flavonoids) were identified and quantified in the ME of the ripe fruits (Table 3). Caffeic acid was the major phenolic acid identified with a concentration of 45.03 ± 0.45 mg/100 g DW. Moreover, HPLC analysis revealed the presence of three flavonoid classes: flavonol, flavone, and flavanone in the ripe fruits. The flavonol class was represented by five compounds, which were quercitrin (52.65 ± 0.31 mg/100 g dried powdered fruits), the major flavonoid detected, followed by kaempferol, then astragalin, quercetin, and rutin. The detected flavones were apigenin and luteolin. While naringenin represented the flavanone class. Other polyphenolic compounds have been found in *F. glaucescens* ripe fruits, highlighting naringenin, apigenin, cinnamic, and *p*-hydroxybenzoic acids as major constituents. Differences in the phenolic profile from different *Ferocactus* fruits may be influenced by genotype, epicarp color, and growing conditions [2, 26]. According to its phenolic profile, this fruit seems suitable as functional food and for pharmaceutical industries.

**Table 3:** Quantifications of some phenolic compounds identified in the fruit methanolic extract (ME) of *F. herrerae* J. G. Ortega using HPLC-UV

Identified PA	Rt (min)	*Conc. (mg /100 g ± SD)	Identified F	Rt (min)	*Conc. (mg /100 g ± SD)
Benzoic acid	2.068	0.93 ± 0.04	Rutin	4.220	1.25 ± 0.01
Gallic acid	4.859	11.77 ± 0.08	Quercitrin	4.614	52.65 ± 0.31
Chlorogenic acid	11.847	19.13 ± 0.20	Astragalin	9.760	1.49 ± 0.22
Caffeic acid	11.55	45.03 ± 0.45	Quercetin	11.460	1.42 ± 0.45
Cinnamic acid	16.292	12.64 ± 0.21	Apigenin	13.512	4.69 ± 0.38
*p*-Coumaric acid	17.807	0.55 ± 0.29	Kaempferol	16.219	36.07 ± 0.45
Sinapic acid	18.721	9.07 ± 0.06	Luteolin	17.228	1.15 ± 0.06
Rosmarinic acid	20.451	0.87 ± 0.09	Naringenin	19.948	1.26 ± 0.21

*Average concentration of three HPLC determinations, F: flavonoids; PA: phenolic acids; Rt; retention time in minutes, SD: standard deviation

### Determination of the Antioxidant Activity

Many threatening pathophysiological conditions such as cardiovascular diseases are related to the accumulation of free radicals in the body. As the innate antioxidant defense system is inefficient, the dietary intake of antioxidants is essential [27]. As a result, the discovery and use of safe antioxidants derived from natural sources are required in the food and medical fields. Since the evaluation of the antioxidant potentials of plant extracts cannot be determined accurately by any single universal method, therefore, we applied several antioxidant assays that would provide a comprehensive picture of the antioxidant potential of *F. herrerae* ripe fruits. The antioxidant activity of the extract was evaluated using free radical scavenging assays (DPPH and ABTS) and the FRAP method.

The free radical-scavenging activity of the ME was 132.06 ± 2.1 and 241.1 ± 5.03 uM TE/g representing 71.11 and 88.41% antioxidant activity compared to ascorbic acid (185.7 ± 1.8 and 272.7 ± 8.2 uM TE/g) in the DPPH and ABTS assays, respectively. Regarding the FRAP assay, the ME exhibited antioxidant activity of 258.91 ± 1.75 *μ*M TE/g representing 89.24% antioxidant activity compared to ascorbic acid (290.1 ± 2.18 uM TE/g). The highest antioxidant capacity of the ME could be attributed to its high content of vitamins and phenolic compounds. Moreover, some of the volatile constituents detected in the fruits as benzyl alcohol (11.69%), linalool (0.65%), and terpineol (0.13%) were reported to possess antioxidant properties [28]. As far as our literature survey could ascertain, this is the first report regarding the DPPH radical scavenging activity of *F. herrarae*. In addition, there is no study concerning the ABTS scavenging ability as well as the FRAP activity of *Ferocactus* species.

### Determination of the Anti-Inflammatory Activity

The ME also showed significant *in-vitro* anti-inflammatory activity. This was evidenced through the significant inhibition of both COX-1 and COX-2 enzymes (IC_50_ = 20.2 ± 1.1 and 9.8 ± 0.64 *μ*g/mL, respectively), when compared to ibuprofen (9.1 ± 0.9 *μ*g/mL) and celecoxib (0.5 ± 0.1 *μ*g/mL), respectively. The results showed a higher selectivity of the ME towards inhibition of COX-2 than COX-1 (SI = 2.06). It has previously been demonstrated that the flavonoids quercetin and naringenin, as well as the phenolic acids rosmarinic and chlorogenic acids, have high binding affinity and good hydrogen bond interactions with the active site residues of both COX-1 and COX-2 [29]. *In silico* experiments also revealed the possible inhibition of COX-1 and COX-2 by quercetin and quercetin derivatives [30]. Furthermore, naringenin isolated from a closely related species (*F. glaucescens*) showed a marked anti-inflammatory activity via inhibition of nitric oxide production [12]. Interestingly, this is the first report of the COX inhibitory activities of *F. herrerae* fruits, which sheds light on their promising health benefits.

### Determination of Acetylcholinesterase Inhibitory (AchEI) Activity

Acetylcholine (Ach), a central and peripheral autonomic nervous system neurotransmitter, plays a key role in the pathophysiology of several diseases [31]. Thus, acetylcholinesterase (AChE) inhibition is one of the useful techniques in the treatment of various conditions including inflammation associated with bacterial infections. In addition, AChE inhibitors (AChEI) are widely used for the treatment of mild to moderate Alzheimer’s disease [32]. Nowadays, many researchers are working to find new AChEI from herbal sources [32]. Thus, we investigated the ability of the fruit extract to inhibit the acetylcholinesterase activity for the first time. Results demonstrated that the ME of *F. herrerae* ripe fruits exhibited significant acetylcholinesterase inhibitory activity with IC_50_ = 1.01 ± 0.39 mg/mL compared to the standard drug (AChEI) physostigmine, with IC_50_ = 0.09 ± 0.1 mg/mL. The observed acetylcholinesterase inhibitory potential of the ME was attributed to its content of compounds of well-reported acetylcholinesterase inhibitory activity. Phenolic acids and flavonoids were previously reported to exhibit acetylcholinesterase inhibitory activity [33]. Quercetin derivatives showed *in silico* acetylcholinesterase inhibitory activity [34]. In addition, the detected vitamins play an important role in delaying the progression of irreversible neurocognitive decline [35]. Overall, it is interesting to note that *F. herrerae* fruits have many biological properties that may recommend their incorporation into the diet, especially for elderly people. The fruits could play an important role in the pathogenesis of Alzheimer’s disease, which is caused by different mechanisms. They may help in the maintenance of acetylcholine levels, enhance cholinergic function, retard neuronal degeneration through their ability to scavenge radical oxygen species, and by attenuating inflammation pathways associated with neuronal degeneration.

## Conclusions

To the best of our knowledge, this is a pioneering report on the nutritional, volatile, and biological profiles of *F. herrerae*, and its first in-depth phytochemical study. The findings herein not only corroborate the significance of the production and consumption of *F. herrerae* fruits, but also revealed the antioxidant, anti-inflammatory, and acetylcholinesterase inhibitory potentials of the ME and correlated them to the high content of phenolic compounds. In this respect, the cited fruits could be recommended as valuable nutritional supplements for the prevention and/or control of serious diseases related to oxidative stress such as Alzheimer’s disease, and inflammatory disorders. However, detailed *in-vivo* and clinical trials are required to validate the identified bioactivities and to facilitate the incorporation of this promising fruit into pharmaceutical formulations.

## Supplementary Information

ESM 1 (DOCX 85.6 mb)

## Data Availability

All data generated or analyzed during this study are included in this published article (and its Supplementary Information file).

## Abbreviations

ABTS: 2,2′-azino-di(3-ethylbenzthiazoline-6-sulfonic acid
AChEI: Acetylcholinesterase inhibitor
COX: Cyclooxygenase enzyme
DPPH: 2, 2-Diphenyl-1-picrylhydrazyl
DW: Dried powdered fruits
FRAP: Ferric reducing antioxidant power
FW: Fresh fruits
GAE: Gallic acid equivalent
HS: Headspace
ME: Fruit methanolic extract
QE: Quercetin equivalent
TE: Trolox equivalent
TFC: Total flavonoid content
TPC: Total phenolic content
TSS: Total soluble solids
