# Supplementary material for: Ferocactus herrerae Fruits: Nutritional Significance, Phytochemical Profiling, and Biological Potentials
Source: Plant Foods Hum Nutr. 2022 Aug 30;77(4):545–51. doi: 10.1007/s11130-022-01007-9 (PMC9606082; doi:10.1007/s11130-022-01007-9)
Supplement: Supplementary file 1 — (DOCX 85.6 mb) [file 11130_2022_1007_MOESM1_ESM.docx]

**Supplementary Material**

*Ferocactus herrerae* Fruits: Nutritional Significance, Phytochemical Profiling, and Biological Potentials

**Passent M. Abdel-Baki^*^**†**, Rana M. Ibrahim**†**, Nariman E. Mahdy**

Pharmacognosy Department, Faculty of Pharmacy, Cairo University, Kasr-El-Ainy Street, 11562, Cairo, Egypt

*****Corresponding author: Passent M. Abdel-Baki, [passent.mohamed@pharma.cu.edu.eg](mailto:passent.mohamed@pharma.cu.edu.eg)

† These authors contributed equally to this work.

**Material and Methods**

**Plant Material**

*Ferocactus herrerae* J. G. Ortega ripe fruits (500 g) were collected during May (2020) from Helal Cactus Farm, Qalyubia Governorate, Egypt. The plant material was authenticated and verified by Botany specialist, Dr. Mohamed El-Gibali, former researcher of Botany, Department of Botany, National Research Centre (NRC). A voucher specimen was deposited in Pharmacognosy Department Herbarium, Cairo University (registration no. 15.1. 2019I). For the examination of the total carbohydrate, total protein, vitamins, and pH of the juice, some fresh rip­e fruits were stored at 4 ^o^C. For evaluation of the crude fibers, minerals, and amino acid composition, part of the ripe fruits was dried in a hot air oven for 24 hours at 45 ^o^C, finely pulverized in a mixer grinder, and then stored in an airtight bottle at 4°C in the refrigerator. For investigation of the fruit volatiles, fresh ripe fruits were collected, finely sliced, and subjected to headspace (HS) extraction. For determination of phenolic content, antioxidant, anti-inflammatory and acetylcholinesterase inhibitory activities, two g of the fresh fruits were homogenized with 100 mL methanol, followed by filtration through Whatman No.1 filter paper, and the solvent was subjected to vacuum evaporation at 50 ^o^C yielding 110 mg of dried residue.

**Chemicals and Reagents**

Folin–Ciocalteu’s reagent was purchased from Loba-Chemie (Mumbai, India). Solvents for HPLC analysis; acetonitrile and methanol were of HPLC grade and were purchased from Sigma–Aldrich (Steinheim, Germany). Distilled water was further purified using a Milli-Q system (Millipore, MA, USA). Acetonitrile and acidulated water were filtered through a 0.45 pm membrane filter (Pall Gelman Laboratory, USA), and degassed in an ultrasonic bath before HPLC analysis. Phenolic acids and flavonoids used in HPLC analysis, 2,2-diphenyl-1-picrylhydrazyl (DPPH), acetylcholinesterase (Electric-eel EC 3.1.1.7), acetylthiocholine iodide (ATCI-substrate), 5,5´-dithiobis[2-nitrobenzoic-acid] (DTNB- chromogen) and all standard drugs were purchased from Sigma. (St. Louis, MO, USA). BioVision’s FRAP Assay Kit (Theo Bhavan Rd, Mavelikara, Kerala 690102, India) and ZenBio ABTS (2,2 azino-bis (3-ethylbenzothiazloine-6-sulfonic acid) Antioxidant Assay Kit (Research Triangle Park, NC 27709, USA) were used for assessment of the antioxidant activity. Cayman colorimetric COX inhibitor screening assay kit was purchased from Cayman Chemical Company (MI, USA). Albumin Bovine serum was obtained from Oxford Lab Chem (Vasai, Maharashtra, India). Tris buffer (50mM) (pH 7.5) was purchased from Biodiagnostic for diagnostic and research reagents (Dokki, Giza, Egypt). Other chemicals used in the study were of analytical grade.

**Nutritional Properties**

**Determination of pH**

Three g of fresh fruits were mixed with 12 mL of deionized water and then measured by a *p*H meter-thermo device.

**Determination of the Total Soluble Solids (TSS)**

One g of fresh fruit was mixed with 1 mL distilled water, then the total soluble solids (TSS was measured by using the refractometer (ATAGO^®^ Pocket PAL-α-Japan). TSS was evaluated in °Brix according to the procedure described by [1].

**Determination of Total Lipid Content**

A rapid soxhlet extraction system (Gerhardt Soxtherm System) was used to determine total lipids (free and conjugated with proteins) according to the procedure of A.O.A.C., 2000 [2].

**Determination of Sugars**

Free sugars were measured spectrophotometrically according to the procedure of Masuko et al*.* [3] using Perkin Elmer Lambda 11 Spectrophotometer (Perkin Elmer, Massachusetts, USA). Glucose, fructose, and sucrose were determined using liquid chromatography with refractive index detection adopting the procedure previously mentioned [4]. The analysis of sugars was performed on HPLC/Agilent 1100, equipped with a G1311A quaternary pump, G1322A degasser, G1329A autosampler, G1330A chiller, G1316A column compartment, ChemStation Software, and a G1382A refractive index detector (RID). A carbohydrate analysis column (Zorbax, 5μm, 150 mm x 4.5 mm Ø) was used at a temperature of 25°C. Isocratic elution using mobile phase was adopted using a mobile phase consisting of acetonitrile: water (75: 25) at a flow rate of 1 ml/min.

**Determination of Vitamins**

Vitamin C and E, and provitamin A contents were determined spectrophotometrically in the fresh samples by adopting the procedure described by Rutkowski et al. [5]. Absorbances were measured at λ= 700, 539, and 335 nm, respectively.

**Determination of Mineral Content**

The mineral content (sodium, calcium, iron, manganese, magnesium, copper, and zinc) was analyzed using Advanced Microwave Digestion System. The different minerals contents were then determined using Inductively Coupled Plasma (ICPA-AES, Thermo Sci, model: ICAP6000 series) [6].

**Determination of Protein and Amino Acid Contents**

Total protein content was estimated as total nitrogen by adopting the method described in A.O.A.C., 2016 [1]. While free amino acids amount was evaluated according to the procedure described previously in Durrum et al. [7]. Free amino acids were measured using an Automatic Amino Acid Analyzer AAA 400 according to the analytical conditions adopted (flow rate, 0.2 mL/min; buffer pressure, 0-50 bar; reagent pressure, 0-150 bar; reactor temperature, 121°C). Free amino acids were determined by comparing the retention times and peak areas of amino acid standards with those of the components present in the sample. The results are displayed in Table 1.

**GC-MS Analysis of Headspace-Extracted Volatiles**

A headspace (HS) autosampler (HSS, 7697 A) was used to monitor the static HS qualitative and quantitative determinations of the volatiles. Five grams were equilibrated for 60 min, at 80°C, prior to analysis. The analysis was performed using an Agilent 7890 GC instrument coupled to a 5977 MS detector and equipped with a silica capillary column, HP-Innovax cross-linked polyethylene glycol adipate (60 m × 0.25 mm × 0.25-μm film thickness). HS autosampler (7697 A) (Agilent Technologies, Palo Alto, CA, USA) was employed to monitor the static HS quantitation of the volatiles. Gas chromatography coupled with mass spectrometry (GC-MS) of the extracted volatiles was carried out on an Agilent 7890 GC instrument coupled to a 5977 MS detector and equipped with a silica capillary column. Adjustment of MS relative abundance for *m/z* 69, 219, and 502 was done manually by tuning using perfluorotributylamine. MS was run in the scan mode (*m/z* range, 33-400; threshold, 100; sampling rate, 3 scans/s). The carrier gas was ultrapure helium passed through moisture and oxygen traps (Hewlett-Packard). The GC operating conditions adopted were flow rate, 1 mL/min at 40°C; split ratio, 1:10; injection port temperature, 250°C; interface line to MS temperature, 230°C; electron energy and electron multiplier voltage at 70 ev and 1647 V. The GC oven temperature program was 40°C for 3 min, 40°C to 180°C at 4°C/min,180°C for 6 min, and increased to 220 °C at 5°C/min. Samples were equilibrated for 60 min at 80°C prior to analysis. The HSS 7697A autosampler settings were 5 s for pressurization, equilibration, and filling, and 2 min for injection. The HS loop (3 mL) temperature was set at 90°C. High-purity helium, filtered through moisture and oxygen traps, was used for vial pressurization, and at a flow rate of 17.5 mL/min measured at the splitter outlet. The linear retention index (RI) values of the constituents were determined based on retention time data obtained by analyzing a series of *n*-alkanes (C_6_-C_22_). Volatile components were identified by matching their RI values and mass spectral data with those of standards run under identical chromatographic conditions [8]. The analysis was run in triplicates. GC-MS chromatogram of the volatile constituents detected in *F. herrerae* ripe fruits was shown in Fig. S1. Components identified, their retention indices (Kovat's indices), molecular ions, relative percentages, and their chemical classes are listed in Table 2.

**Spectrophotometric Determination of Phenolic Compounds**

**Total Phenolic Content (TPC)**

The total phenolic content (TPC) in the fruit methanolic extract (ME) was determined in triplicates using the Folin–Ciocalteu method according to the procedures described before [9] using Unicam UV–visible Spectrometer. Gallic acid was used to establish the standard calibration curve. The results were presented as mean values with standard deviations in mg gallic acid equivalent (GAE) per gram of dry extract (DW).

**Total Flavonoid Content (TFC)**

The total flavonoid content (TFC) was determined in triplicates using the aluminum chloride method [10] adapted to a plate reader. Quercetin was used to compute the standard calibration curve. The results were reported as mean values with standard deviations in mg quercetin equivalent (QE) per gram of dry extract (DW).

**Quantitative Determination of Phenolic Compounds by HPLC-UV**

Quantitative determination of phenolic compounds was performed using high-performance liquid chromatography (HPLC) apparatus, Agilent Series 1200 apparatus (Agilent, USA) consisting of autosampler injector, solvent degasser, quaternary HP pump (series 1200), 1100 ChemStation software, and ultraviolet (UV) detector (set at 280 nm for phenolic acids and 330 nm for flavonoids). The analysis was achieved on a Zorbax ODS C18 column (particle size 5 µm, 250 mm × 4.6 mm Ø). Flavonoid separation was performed according to the method described previously [11]. Gradient elution with a mobile phase consisted of A (50 mM H_3_PO_4_, pH 2.5) and B (acetonitrile: acetic acid (40:60, v/v)) in the following order: 0–5 min; linear gradient from 95% A: 5% B to 50% A: 50% B, 5–55 min; isocratic elution of 50% A: 50% B, 55–65 min; linear gradient from 50% A: 50% B to 95% A: 5% B, 65–67 min; was used. The solvent flow rate was 0.7 ml/min, and the separation was performed at 35°C.

Phenolic acid separation was performed by adopting the procedure of Goupy et al*.* [12]. The solvent system consisted of A (aqueous acetic acid 2.5%), B (aqueous acetic acid 8%) and C (acetonitrile) in the following gradient: at 0 min, 5%B; at 20 min, 10% B; at 50 min, 30% B; at 55 min, 50% B; at 60 min, 100% B; at 100 min, 50% B and 50% C; at 110 min, 100% C until 120 min. The solvent flow rate was 1 ml/min and the injection volumes were 5 µL. Standard flavonoids and phenolic acids were prepared as 10 mg/50 ml solutions in methanol and diluted to make concentrations ranging from 20–40 g/ml, then injected into HPLC. Concentrations of the compounds were determined depending on peak area computation (external standard method). The analysis was run in triplicates and HPLC chromatograms were illustrated in Fig. S2 and Fig. S3. The concentrations of the identified compounds were expressed as mg ± SD/100 g dry weight (DW) and recorded in Table 3.

**Determination of the Antioxidant Activity**

**DPPH Radical-Scavenging Activity**

The DPPH free radical-scavenging activity of the ME was assessed according to the previously reported method [13] with slight modifications using a SPECTROstar Nano microplate reader (BMG Labtech, Offenburg, Germany). The experiments were performed in triplicates and the results were expressed as mean ± SD. Ascorbic acid was used as a standard antioxidant. A standard stock solution of Trolox (6-hydroxy-2,5,7, 8-tetramethylchroman-2-carboxylic acid) (600 μM) was serially diluted with the assay buffer to give the required concentrations equivalent in the range of 4.6875-300 μM. The results were expressed as Trolox equivalent (TE) per gram of dry extract (TE/g).

**ABTS Radical-Scavenging Activity**

The scavenging capacity of the ME against 2, 2’-azino-bis (3-ethylbenzthiazoline-6-sulfonic acid) (ABTS) assay was measured according to the manufacturer’s instructions as well as the reported method [14]. ABTS radical cation (green in color) was produced due to oxidation of ABTS by ferrylmyoglobin radical that is formed from metmyoglobin (present in the chromogen provided in the kit) and hydrogen peroxide. Antioxidants suppress this reaction by electron donation radical scavenging and thus prevent the formation of the colored ABTS radical. The concentration of antioxidants in the tested sample is inversely proportional to the ABTS radical formation. Ascorbic acid was used as standard antioxidant. Results were expressed as micromoles (μM) Trolox equivalent (TE) per gram of tested sample (i.e., μM TE/g). Triplicate measurements were done for the tested sample.

**Ferric Reducing Antioxidant Power (FRAP) Assay**

FRAP assay is a method that employs antioxidants as reductants in a redox-linked colorimetric reaction, wherein ferric (Fe^3+^) is reduced to ferrous (Fe^2+^) at low pH, resulting in the formation of a blue-colored ferrous-probe complex from a colorless ferric-probe complex. The absorbance of the blue color produced is measured at λ 594 nm. This procedure was carried out to measure the FRAP of the fruit methanolic extract (ME) according to the manufacturer’s instructions, and as indicated in former publications [15]. Ascorbic acid was used as standard antioxidant. Results were expressed as μM TE after the construction of Trolox calibration curve. Triplicate measurements were carried out for each sample or standard.

**Determination of the Anti-inflammatory Activity**

The anti-inflammatory potential of *F. herrerae* ME was investigated by inhibiting the cyclooxygenases (COX-1 and COX-2) enzymes. COX-1 and COX-2 inhibitory activities were measured according to the procedures described previously [16, 17] with slight modifications. The ME and the standard drugs (ibuprofen and celecoxib for COX-1 and COX-2, respectively) were prepared in dimethylsulfoxide (DMSO) at the concentration range of 125- 0.98 µg /mL in a 96-well plate. The inhibitory COX activity was determined by monitoring the absorbance’s increase at 611 nm, as the result of the N,N,N,N-tetramethyl-p-phenylenediamine (TMPD) oxidation reaction with arachidonic acid. Fifty μL of the enzyme (200 units of COX-1 or COX-2/cuvette) and 100 μL of co-factor (3M hematin in Tris-buffer, pH 8) \ and 1000 μL of buffer (100mM Tris–HCl buffer, pH 8) and 50 μL of DMSO per sample were pre-incubated. This enzyme/co-factor solution was added to the test solution consisting of 50 μL of each tested sample concentration and pre-incubated for 3 min at 25 ◦C. Then 200 μL of TMPD was added to each well. To initiate the reaction, 50 μL of arachidonic acid was added to the enzyme/extract mixture, and the contents were mixed immediately. The inhibitory percentages were calculated according to the formula:

Inhibitory activity (%) = (1 – As/Ac) ×100

where, As is the absorbance in the presence of test substance and Ac is the absorbance of control.

The efficacy of the extract and the standard drugs to inhibit COX-1 and COX-2 was determined as the concentration causing 50% enzyme inhibition (IC_50_). Triplicate determinations were performed for each tested sample concentration and the standards. The selectivity index (S.I.) was calculated as IC_50_ (COX-1)/IC_50_ (COX-2).

**Determination of Acetylcholinesterase Inhibitory (AchEI) Activity**

The acetylcholinesterase inhibitory (AchEI) activity of *F. herrerae* ME was measured according to a modified Ellman′s method [18]. A set of serial dilutions of the methanolic extract was established. Acetylthiocholine iodide (ATCI) was used as acetylcholinesterase substrate. Ellman’s reagent (5,5´-Dithiobis[2-nitrobenzoic-acid] (DTNB) was used for the measurement of cholinesterase activity. The reaction mixture for each determination consisted of DTNB (125 μL, 0.08mM) in tris buffer (pH= 7.5), tested sample in methanol (25 μL), ATCI (25 μL, 0.4 mM) in distilled water and acetylcholinesterase (AchE) (25 μL) in Tris buffer (pH = 7.5) having 0.1% bovine serum (0.06 U/mL). The hydrolysis of acetylthiocholine was determined by the formation of 5- thio-2-nitrobenzoate anion (yellow colour) as the result of the reaction of DTNB with thiocholine, released by the enzymatic hydrolysis of acetylthiocholine at a wavelength of 405 nm. The rate of AchE hydrolysis was measured over 15 min using a 96- well microplate reader and triplicates were done for each dilution. A blank experiment was carried out using 25 μL Tris buffer instead of the sample (Ablank_1_). In the same way, the enzyme’s 100 percent activity was assessed using 25 μL of methanol instead of the sample (Aenzyme), and the enzyme’s blank was assessed using 25 L of Tris buffer (pH=7.5) instead of the enzyme (Ablank_2_). A stock solution of physostigmine was made by dissolving 2 mg of the standard in 1 mL methanol, followed by serial dilutions to yield concentrations ranging from 7.8 to 2000 μg/mL. The standard was treated as described above. The AchEI activity was determined using the following equation:

Percentage of inhibition = 100 [1-((Asample-Ablank1)/(Aenzyme-Ablank2))]

Where, Asample = Average sample absorbance

Ablank1 = Average sample blank absorbance

Aenzyme = Average enzyme activity without tested sample absorbance

Ablank2 = Average of the Aenzyme blank

The IC_50_ values (inhibitory concentration of the sample causing 50% inhibition of AchE activity) were determined using GraphPad Prism (version 5.01, Inc., 2007, San Diego California USA) software program. The AchEI activity of the extract was compared to that of physostigmine. Triplicate measurements were done for each tested sample concentration and standard.

**Statistical analysis**

The data of all the experiments performed were expressed as mean values and standard deviations

(SD). All the experiments were performed in triplicate (n=3). Linear regression was performed for calculation of 50% inhibitory concentration (IC_50_). To determine the differences between a sample and the corresponding standard, Student’s t-test was used. Differences were considered significant if the p values were <0.05. Data analysis was performed using Microsoft Excel 2016 program and GraphPad Prism Version 6.00.

**References**

1. Latimer GWJ, International A, Cunniff P (2016) Official Methods of Analysis of AOAC International. AOAC International

2. Horwitz W, Chemists AoOA (2000) Official methods of analysis of the AOAC. Association of Official Analytical Chemists

3. Masuko T, Minami A, Iwasaki N, Majima T, Nishimura S-I, Lee YC (2005) Carbohydrate analysis by a phenol–sulfuric acid method in microplate format. Anal Biochem 339(1):69-72. https://doi.org/[10.1016/j.ab.2004.12.001](https://doi.org/10.1016/j.ab.2004.12.001)

4. Varandas S, Teixeira MJ, Marques JC, Aguiar A, Alves A, Bastos MMSM (2004) Glucose and fructose levels on grape skin: interference in *Lobesia botrana* behaviour. Anal Chim Acta 513(1):351-355. <https://doi.org/10.1016/j.aca.2003.11.086>

5. Rutkowski M, Grzegorczyk K (2007) Modifications of spectrophotometric methods for antioxidative vitamins determination convenient in analytic practice. Acta Sci Pol Technol Aliment 6(3):17-28

6. Kira CS, Maio FD, Maihara VA, Maihara VA (2004) Comparison of partial digestion procedures for determination of Ca, Cr, Cu, Fe, K, Mg, Mn, Na, P, and Zn in milk by inductively coupled plasma-optical emission spectrometry. J AOAC Int 87(1):151-156

7. Durrum EL, Block RJ, Zweig G (1958) A manual of paper chromatography and paper electrophoresis. Academic Press

8. Adams RP (2007) Identification of essential oil components by gas chromatography/mass spectrometry. Allured publishing corporation Carol Stream

9. Shweta S, Ritesh T, Khadabadi SS, Deokate UA (2010) *In vitro* antioxidant activity and total phenolic, flavonoid contents of the crude extracts of *Pterospermum acerifolium* Willd leaves (Sterculiaceae). J Chem Pharm Res 2(3):417-423

10. Kiranmai M, Kumar C, Ibrahim M (2011) Comparison of total flavanoid content of *Azadirachta indica* root bark extracts prepared by different methods of extraction. Res J Pharm Biol Chem Sci 2(3):254-261

11. Mattila P, Astola J, Kumpulainen J (2000) Determination of flavonoids in plant material by HPLC with diode-array and electro-array detections. J Agric Food Chem 48(12):5834-5841. <https://doi.org/10.1021/jf000661f>

12. Goupy P, Hugues M, Boivin P, Amiot MJ (1999) Antioxidant composition and activity of barley (*Hordeum vulgare*) and malt extracts and of isolated phenolic compounds. J Sci Food Agric 79(12):1625-1634.

13. Martins N, Barros L, Dueñas M, Santos-Buelga C, Ferreira IC (2015) Characterization of phenolic compounds and antioxidant properties of *Glycyrrhiza glabra* L. rhizomes and roots. RSC Adv 5(34):26991-26997. <https://doi.org/10.1039/C5RA03963K>

14. Mocan A, Schafberg M, Crișan G, Rohn S (2016) Determination of lignans and phenolic components of *Schisandra chinensis* (Turcz.) Baill. using HPLC-ESI-ToF-MS and HPLC-online TEAC: Contribution of individual components to overall antioxidant activity and comparison with traditional antioxidant assays. J Funct Foods 24:579-594. https://doi.org/[10.1016/j.jff.2016.05.007](http://dx.doi.org/10.1016/j.jff.2016.05.007)

15. Damiano S, Forino M, De A, Vitali LA, Lupidi G, Taglialatela-Scafati O (2017) Antioxidant and antibiofilm activities of secondary metabolites from *Ziziphus jujuba* leaves used for infusion preparation. Food Chem 230:24-29. https://doi.org/[10.1016/j.foodchem.2017.02.141](https://doi.org/10.1016/j.foodchem.2017.02.141)

16. Amessis-Ouchemoukh N, Madani K, Falé PL, Serralheiro ML, Araújo MEM (2014) Antioxidant capacity and phenolic contents of some Mediterranean medicinal plants and their potential role in the inhibition of cyclooxygenase-1 and acetylcholinesterase activities. Ind Crops Prod 53:6-15. https://doi.org/[10.1016/j.indcrop.2013.12.008](http://dx.doi.org/10.1016%2Fj.indcrop.2013.12.008)

17. Petrovic N, Murray M (2010) Using N, N, N’, N’-tetramethyl-p-phenylenediamine (TMPD) to assay cyclooxygenase activity *in vitro*. Advanced Protocols in Oxidative Stress II, Springer, pp 129-140

18. Mathew M, Subramanian S (2014) *In vitro* screening for anti-cholinesterase and antioxidant activity of methanolic extracts of ayurvedic medicinal plants used for cognitive disorders. PloS one 9(1):e86804. https://doi/org/ [10.1371/journal.pone.0086804](https://doi.org/10.1371/journal.pone.0086804)

**Supplementary Tables**

**Supplementary Figures**

|  |
| --- |
| Fig. S1 Total ion chromatogram of the headspace-extracted volatiles of *F. herrerae* ripe fruits |
| \|  \| \| --- \| \| Fig. S2 HPLC chromatogram showing identified phenolic acids in the fruit methanolic extract (ME) of *F. herrerae* J. G. Ortega measured at 280 nm \| \|  \| \| Fig. S3 HPLC chromatogram showing identified flavonoids in the fruit methanolic extract (ME) of *F. herrerae* J. G. Ortega measured at 330 nm \| |
